# Dysbiosis and unsustainable delayed gut microbiota development as non-invasive biomarkers for predicting autism spectrum disorder in Chinese children

**DOI:** 10.3389/fmicb.2026.1753665

**Published:** 2026-06-18

**Authors:** Hui Li, Ningshan Li, Chengju Wang, Jun Yang, Zongming Dong, Zhiping Cai, Jia Li, Yu Chen, Ji Zheng, Jingzhen Zhu

**Affiliations:** 1 School of Life Sciences and Technology, Tongji University, Shanghai, China; 2 Department of Ultrasound, Second Affiliated Hospital, Army Medical University, Chongqing, China; 3 Department of Pediatric, Second Affiliated Hospital, Army Medical University, Chongqing, China; 4 Department of Urology, Second Affiliated Hospital, Army Medical University, Chongqing, China

**Keywords:** ASD, biomarkers, ecological network, gut microbiota, microbial-gut-brain axis

## Abstract

**Introduction:**

Autism spectrum disorder (ASD) is a neurodevelopmental disorder characterized by social impairment, restricted interest, repetitive behavior, and stereotypical behavioral patterns. Diagnosing ASD presents considerable challenges; a previous large-sample study in children linked ASD and intestinal flora imbalances.

**Methods:**

To explore the composition and functional changes of the gut microbiota in children with ASD, shotgun metagenomic sequencing was used to evaluate the gut microbiota of 78 Chinese children (34 with ASD and 44 with typical development [TD] children).

**Results:**

We observed differences in the gut microbiota composition and richness between children with ASD and TD in this cohort. The α-diversity of the gut microbiota in the ASD group fluctuated more with age than that in the TD group, based on cross-sectional data. Age-related dynamic changes in the gut bacteria of TD children were not clearly observed in children with ASD. Gut microbiota of children with ASD showed a higher number of antibiotic resistance genes compared to TD. Additionally, the functional gene pathways related to carbohydrate-active enzymes and amino acid metabolism and synthesis appeared reduced in the ASD group.

**Discussion:**

This exploratory study describes key compositional and functional characteristics of the gut microbiota in Chinese children with ASD. Our preliminary findings identify differential bacterial taxa that may be considered as potential candidates for further investigation as fecal markers, and suggest differences in age-related gut microbiota patterns between ASD and TD children. However, due to the modest sample size, cross-sectional design, and lack of external validation, these results should be regarded as a preliminary exploration and require confirmation in larger, independent cohorts.

## Introduction

Autism spectrum disorder (ASD) is a neurodevelopmental disorder characterized by social and language communication deficits and repetitive stereotypical behaviors (Rosen et al., 2021). Although genetic factors contribute to ASD, they explain only a small proportion of cases, and accumulating evidence points to environmental factors, particularly the gut microbiota, in its etiology (Wan et al., 2022). Gastrointestinal problems are common non-neurological manifestations in children with ASD (Ferguson et al., 2019).

The gut-microbiota-brain axis modulates brain development and behavior through immune regulation, microbial metabolites, and neurotransmitter production (Agirman and Hsiao, 2021). Animal models have shown that transplanting ASD-derived gut microbiota induces ASD-like behaviors in mice, and that specific microbial interventions can reverse social deficits (Davies et al., 2021; Sharon et al., 2019); Clinically, children with ASD frequently experience gastrointestinal symptoms that correlate with ASD severity, and our previous large-sample study demonstrated significant differences in gut microbiota diversity, structure, and relative abundance between ASD and typically developing (TD) children (Li et al., 2023). Moreover, bacterial α-diversity changes with age in ASD, suggesting a persistent delay in gut microbiota development compared with age-matched peers (Ahrens et al., 2024; Wan et al., 2022).

Despite these advances, most previous microbiome studies have used 16S rRNA sequencing, which lacks species-level and functional resolution. Furthermore, ASD has often been treated as a homogeneous condition across wide age ranges, with rare stratification by age or gastrointestinal symptoms. Consequently, the potential of specific gut bacterial species as non-invasive biomarkers for ASD remains underexplored, and the developmental trajectory of the gut microbiota in ASD has not been systematically evaluated. To address these knowledge gaps, we performed shotgun metagenomic sequencing (enabling species-level and functional gene module resolution) on fecal samples from Chinese children with ASD and TD controls. We aimed to explore candidate microbial species as potential non-invasive ASD biomarkers and to assess gut microbiome developmental patterns in ASD. This exploratory study offers preliminary insights that may inform future diagnostic or interventional approaches, but further validation is required.

## Materials and methods

### Ethics statement

This study was a retrospective study, and the authors assert that all procedures contributing to this work complied with the ethical standards of the relevant national and institutional committees on human experimentation and with the Declaration of Helsinki. The Ethics Committee of the Second Affiliated Hospital of the Army Medical University approved this study (Ethics No. 2023-001-01). The research process will not cause any physical or psychological harm to minors, nor will it disclose their sensitive personal information. Stringent data encryption and anonymization measures have been implemented. According to relevant theories in psychology and pedagogy, the minors involved in this study fall within the age range of 3–12 years old. At this stage, children's cognitive and decision-making abilities are not yet fully developed, and they may not fully comprehend the nature, purpose, and potential implications of the research. To safeguard the rights and interests of minors, we have adhered to internationally recognized ethical guidelines and decided that parents/guardians will exercise the right of consent on their behalf, and we obtained verbal informed consent from the parents/guardians. The trial was registered in the Chinese Clinical Trial Registry (www.chictr.org.cn; trial registration number: ChiCTR2300074832).

### Study cohort description

In all, 78 participants were included, of whom 34 were children with clinically defined ASD (aged 3–12 years) and 44 with TD (aged 3–12 years). The male:female ratios were 2.4:1 and 6.3:1 in the ASD and TD groups, respectively. Detailed information is provided in Table 1.

**Table 1:** Clinical information of the study participants.

Characteristic	ASD	TD
Total participants (*n*)	34	44
Age range (years)	3–12	3–12
Average age (years)	6.32	6.70
Male/female	24/10	38/6
Clinical feature	Previous diagnosis of ASD	Community health control

ASD, autism spectrum disorders; TD, typically developing.

Diagnosis of all children with ASD participating in this study was according to the Diagnostic and Statistical Manual for Mental Disorders, fifth Edition criteria. TD children were selected from the community as controls. Children with mental illnesses such as depression, schizophrenia, and bipolar disorder based on entrance exams and parent interviews were excluded. None of the participants had taken antibiotics within 1 month before samples were collected. Stool samples were collected from participants in hospitals or at home and immediately frozen. Frozen stool samples were transported overnight on dry ice to CNNC Yili (Tianjin) Medical Laboratory Co., Ltd., where they were frozen at −80 °C until the DNA was extracted.

### Fecal DNA extraction and library construction

Fecal microbial DNA was extracted using a fecal DNA kit (DP328, TIANGEN, China). The sequencing library was constructed using Illumina's FS Pro DNA Lib Prep Kit (RK20261, ABclonal, USA). The constructed library was used for ultra-deep metagenomic sequencing on a DNBSEQ-T7 Sequencer (MGI, HuaDa, China) using the sequencing strategy PE150, and the amount of data per sample was 6G. Sequence data associated with this project has been saved in the National Genomics Data Center (Nuclear Acids Res 2022; GSA: CRA013248), is of public access, and can be found in https://bigd.big.ac.cn/gsa/browse/CRA013248.

### Data processing and analysis

Shotgun metagenomic sequencing was performed using the MGI DNBSEQ-T7 (PE 150 bp). Raw reads were filtered to remove low-quality bases (*Q* < 20) and adapter sequences using fastp (v0.23.2). Readfq (https://github.com/cjfields/readfq) was used to read and convert sequence formats after fastp filtering. Bowtie2 (http://bowtie-bio.sourceforge.net/bowtie2/index.shtml) was used to filter out reads from the host. Assembly analysis of Clean Data was carried out through MEGAHIT. ORF prediction of Scaftigs (≥500 bp) from each sample was performed using MetaGeneMark (http://topaz.gatech.edu/GeneMark/) with default parameters. CD-HIT (http://cd-hit.org/) software was used to remove redundancy from the ORF prediction results to obtain a non-redundant initial gene directory.

Bowtie2 was used to compare the clean data of each sample with the initial gene directory and calculate the number of reads on each sample alignment. Genes with reads ≤ 2 in each sample were excluded to obtain the final gene directory for subsequent analysis (Unigenes). Unigene sequences were aligned against bacterial, fungal, archaeal, and viral sequences in the NR database (https://www.ncbi.nlm.nih.gov/) of NCBI using DIAMOND (https://github.com/bbuchfink/diamond/).

Based on abundance tables at each classification level, relative abundance profile analysis, and abundance clustering heatmap analysis were conducted, and principal co-ordinate analysis (PCOA, R vegan package) and non-metric multidimensional scaling (NMDS, R vegan package) dimensionality reduction analysis were performed. Analysis of similarities (ANOSIM) was used to test the differences between groups. The between-group LEfSe differential analysis first employed a non-parametric Kruskal–Wallis test to identify variations in species abundance among different groups, identifying species that exhibit significant differences. It then utilized the Wilcoxon rank-sum test to assess the consistency of these differences within different subgroups between groups. Finally, linear discriminant analysis (LDA) was used to estimate the influence of these different species on group distinctions. Finally, Random forest (RF) analysis was performed using species-level relative abundance data. The dataset was randomly divided into a training set (80% of samples) and a test set (20% of samples). The RF model was built on the training set using the randomForest package in R (ntree = 500, mtry = default). Feature importance was evaluated by MeanDecreaseAccuracy and MeanDecreaseGini. To avoid overfitting, a five-fold cross-validation was applied within the training set. The final model was then applied to the test set, and receiver operating characteristic (ROC) curves were plotted to calculate the area under the curve (AUC). The AUC value from the test set was reported to indicate the predictive performance.

Next, gene function analysis was performed. The Kyoto Encyclopedia of Genes and Genomes (KEGG) database (https://www.kegg.jp/) was used for metabolic pathway analysis, CARD database (https://card.mcmaster.ca/) for resistance genes analysis, eggNOG database (http://eggnog6.embl.de/) for orthologous genes analysis, and CAZy database (http://www.cazy.org/) for carbohydrate enzyme function analysis.

### Co-occurrence network analysis

A co-occurrence network was constructed based on shotgun metagenomic sequencing data to understand the correlations among the different genera (Wang et al., 2018). Based on the relative abundance of each genus, bacterial correlations in ASD and TD samples were analyzed using Spearman's correlation coefficient to construct co-occurrence networks. Gephi 0.9.7 (www.netbeans.org) was used to identify significant related genera (FDR *p* < 0.05; *r* ≥ 0.7). Node proximity and shared correlation were used to measure the similarity between two network structures. Gephi was used to analyze the closeness of nodes and predict the node centrality of each network. Edges with the same nodes in two co-occurrence networks were defined as shared associations between the two groups.

### Statistical analysis

All data are expressed as mean ± standard deviation. Statistical comparisons between two measurements were performed using SPSS software (v26.0; IBM Corp., Armonk, NY, USA) using independent samples *t*-tests. A two-tailed Wilcoxon rank-sum test was performed to analyze the gut microbiota sequencing data using R software. Geographical region was included as a covariate in linear mixed-effects modeling (Viechtbauer, 2010) to mitigate potential confounding effects associated with geographical heterogeneity. Statistical significance was set at *p* < 0.05 or *q* < 0.05.

## Results

**Figure 1:**
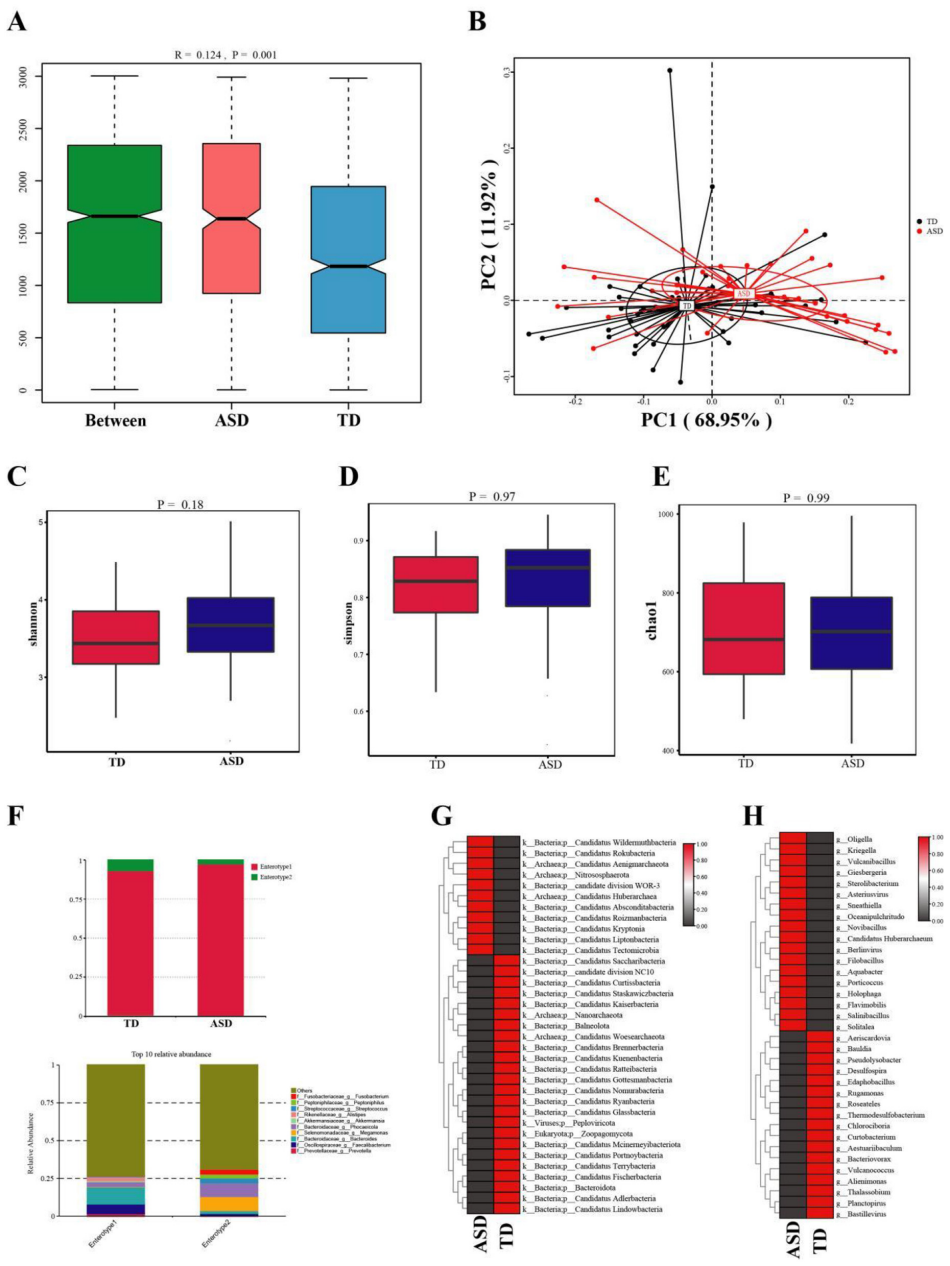
Alterations in gut microbiome in Chinese children with autism spectrum disorder (ASD). **(A)** Analysis of similarity (ANOSIM) analysis, an *R*-value greater than 0 indicates that the inter-group difference is greater than the intra-group difference, with larger *R*-values indicating larger inter-group differences. “Between” represents the distance/rank distribution between samples from different groups (ASD vs. TD). An *R*-value less than 0 indicates that the intra-group difference is greater than the inter-group difference, with a value closer to −1 indicating a greater intra-group difference. A smaller *p*-value indicates a higher significance of differences between sample groups. *p* < 0.05 indicates statistical significance. **(B)** Principal co-ordinate analysis (PCoA) of bacterial community composition in the ASD and typically developing (TD) group, PCoA is plotted based on the selected distance matrix. **(C–E)** Alpha diversity based on the Shannon, Simpson and Chao1 index. **(F)** Enterotype analysis of the gut microbiome in the ASD and TD groups. **(G, H)** Heatmap analysis of the relative abundance log values of ASD group and TD group at the level of phylum and genus (*p* < 0.05), where colors represent the magnitude of relative abundance log values of the microbiota, with red indicating high relative abundance and black indicating low relative abundance.

**Figure 2:**
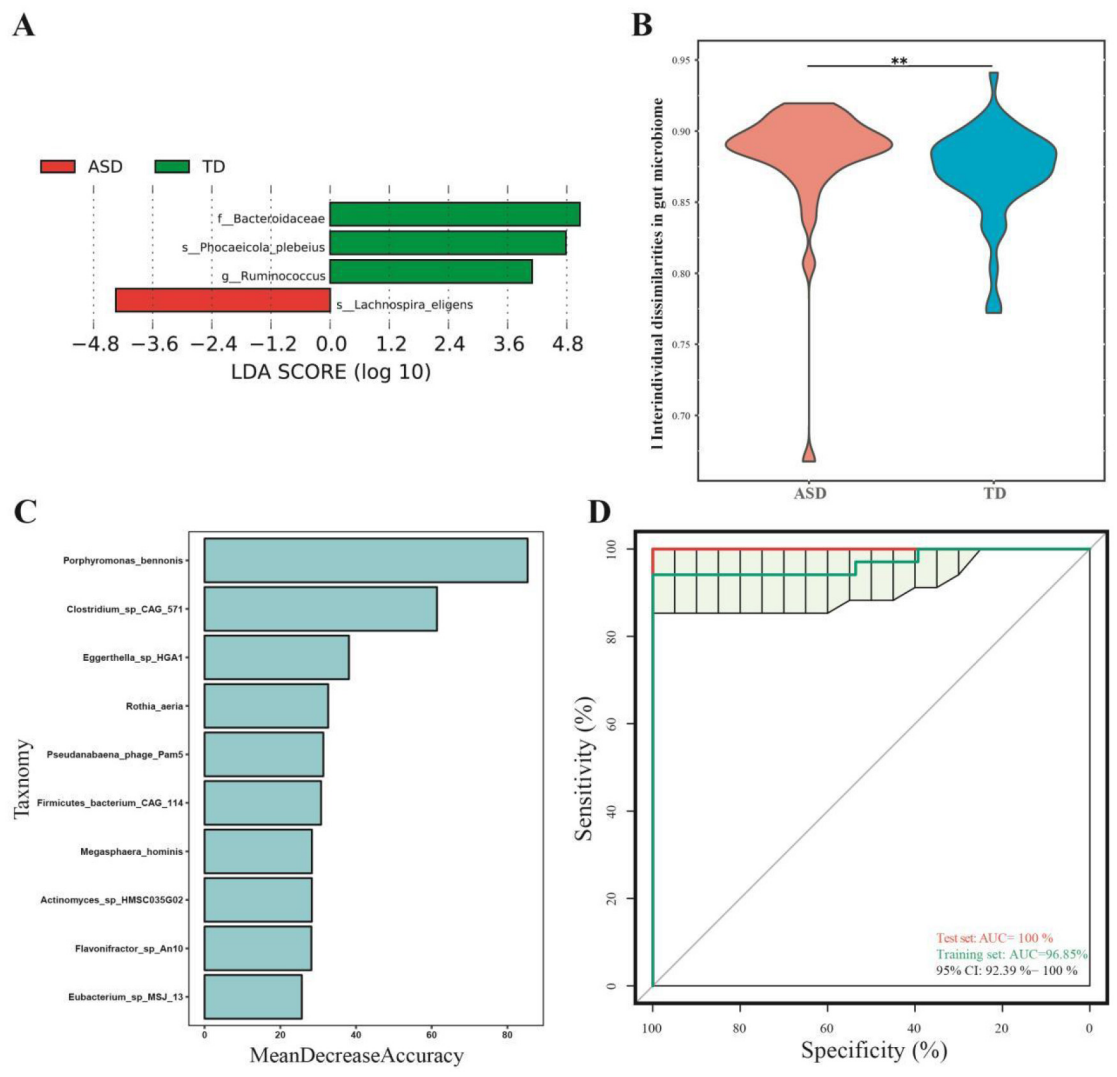
Biomarkers of differential gut microbiota between children with autism spectrum disorder (ASD) and typically developing (TD) children. **(A)** The difference in the relative abundance of the gut microbiota using LEfSe (*p* < 0.05; *LDA* > 3.5). **(B)** Interindividual dissimilarities between the gut microbiomes within each group. The microbiome dissimilarity was calculated as Bray–Curtis dissimilarity. Between-group comparison was conducted by *t*-test. **(C)** The 10 species used to classify ASD and TD were obtained by analyzing the relative abundance of gut microbiota species using random forest regression. **(D)** Random forest classifier performance for classifying ASD vs. TD microbiomes. The area under the curve (AUC) values of the training and test sets are represented by the **green** and **red** lines, respectively. *p < 0.05, **p < 0.01, and ***p < 0.001.

**Figure 3:**
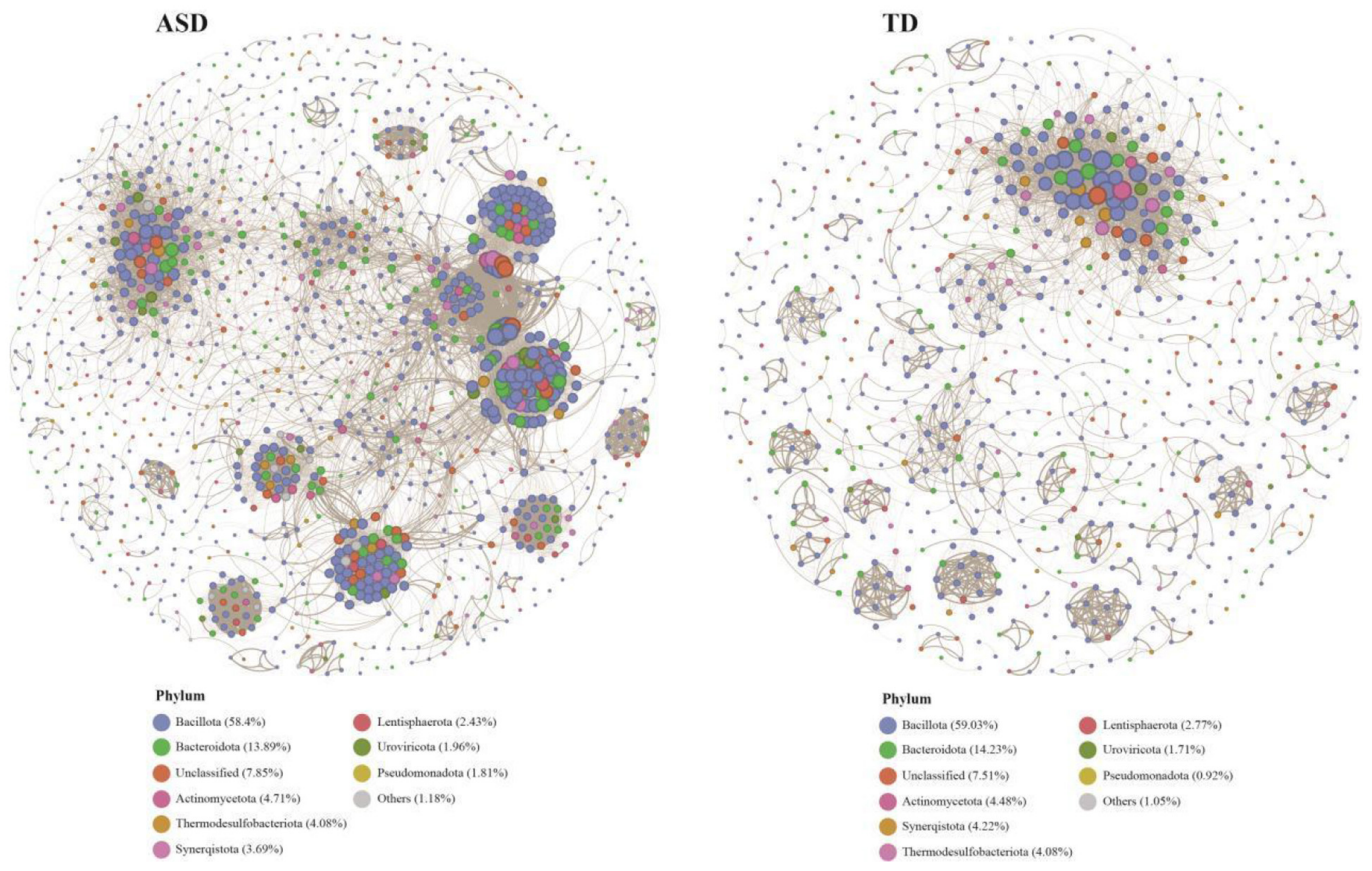
Gut bacterium ecological network in children with autism spectrum disorder (ASD) vs. typically developing (TD) children. General co-occurrence network between the ASD and TD groups based on Spearman's correlation algorithms. Each node represents a bacterial genus. Node size indicates the weighted value of each genus per group, and the color of the node indicates the phylum to which the corresponding microbial species belong. The thickness of the line represents Spearman's coefficient (*p* < 0.05; *r* ≥ 0.7).

**Figure 4:**
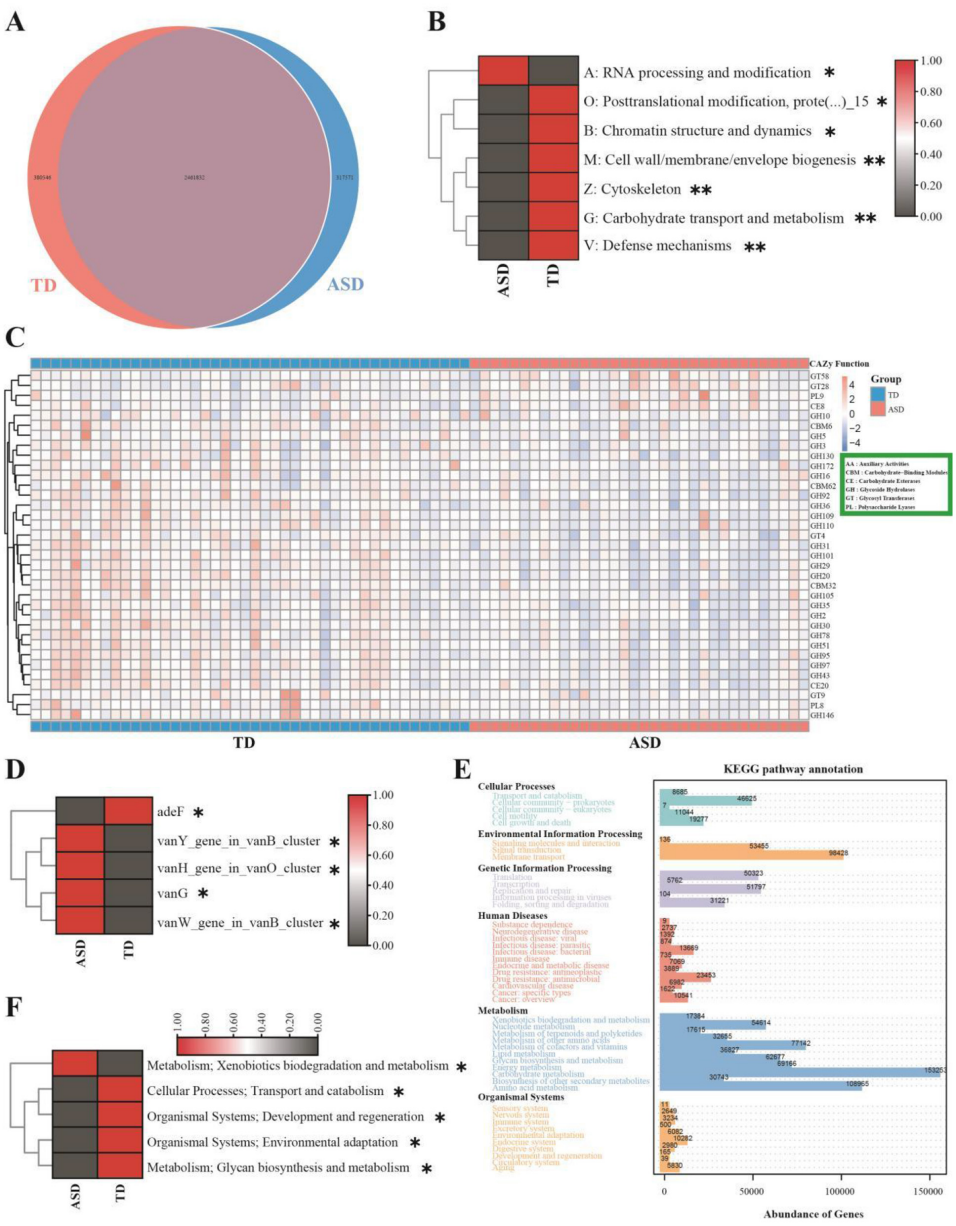
Functionality alterations in the gut microbiome in children with autism spectrum disorder (ASD). **(A)** Venn diagram of gut microbial genes in the ASD and typically development (TD) groups. **(B)** Functional analysis of direct homologous genes in children with ASD and TD children based on the Smith–Waterman alignment algorithm. Color represents the relative abundance log value of the function, with red representing high enrichment abundance and black representing low enrichment abundance. **(C)** Heatmap analysis of the significant differences in CAZymes between children with ASD and TD children, Cluster according to the functional richness value, with color depth indicating the degree of functional enrichment. **(D)** Significant differences in drug resistance genes between children with ASD and TD children; colors represent the relative abundance log values of drug-resistant genes, with red indicating high enrichment abundance and black indicating low enrichment abundance. **(E)** Comparison of the main Kyoto Encyclopedia of Genes and Genomes (KEGG) functions. **(F)** Significant differences in KEGG metabolic pathways in children with ASD and TD children; color represents the relative abundance log value of KEGG functions, with red indicating high enrichment abundance and black indicating low enrichment abundance. *p < 0.05, **p < 0.01, and ***p < 0.001.

**Figure 5:**
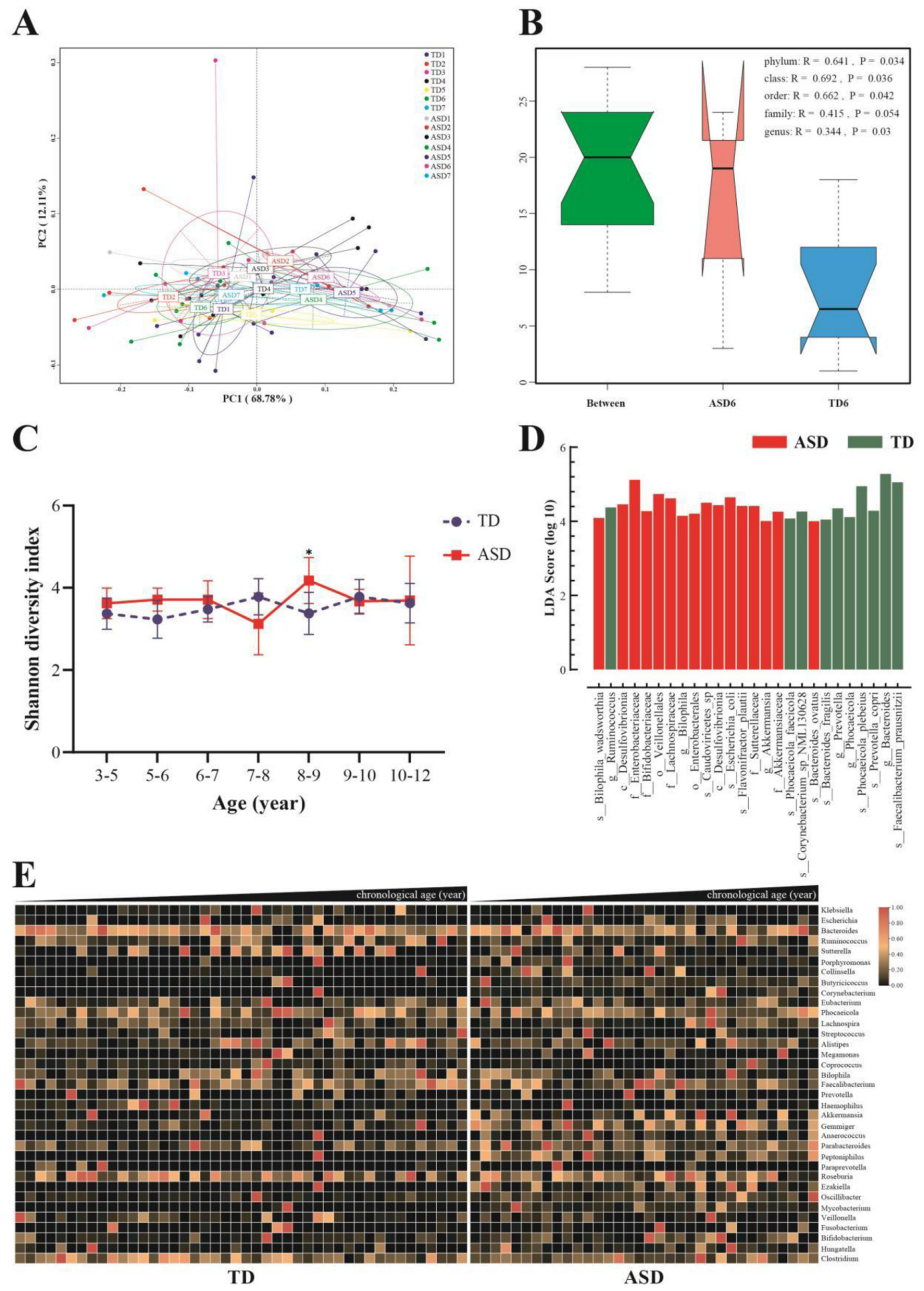
Gut microbiota development is delayed in children with autism spectrum disorder (ASD). **(A)** Principal coordinate analysis based on the gut microbiota age in children with ASD and typical development (TD). **(B)** ANOSIM analysis at different taxonomic levels; an *R*-value greater than 0 indicates that the differences between groups are greater than within groups, with *R*-values closer to 1 indicating greater between-group differences. An *R*-value less than 0 indicates that within-group differences are greater than between-group differences, with *R*-values closer to −1 indicating greater within-group differences. A smaller *p*-value indicates a higher significance of differences between sample groups, with *p* < 0.05 indicating statistical significance. **(C)** Changes in the Shannon diversity index over time in children with ASD and TD children. **(D)** Difference in the relative abundance of gut microbiota using LEfSe (*p* < 0.05; *LDA* > 3.5). **(E)** Heatmap of the top 35 relative abundance of the gut microbiota plotted against the chronological age spectrum (years) in TD children and children with ASD.

1. **Gut microbiota composition in Chinese children with ASD and TD**
  We compared the gut microbial profiles between children diagnosed with ASD and TD children. Analysis of similarities (ANOSIM) showed that inter-group variation exceeded intra-group variation, presenting a statistically significant grouping effect (*r* = 0.124; nominal *p* = 0.001; Figure 1A). Principal coordinate analysis (PCoA) based on the genus level displayed showed that PC1 and PC2 explained 68.95% and 11.92% of the variability, respectively. The PCoA plot exhibited partial overlap with a trend toward separation of samples between the ASD and TD groups (Figure 1B). At the genus level, α-diversity analysis showed that the diversity of intestinal microbiota was higher in the ASD group than in the TD group; however, the difference was not statistically significant (Shannon *p* = 0.18, Simpson *p* = 0.97, Chao1 *p* = 0.99; Figures 1C–E). Enterotype analysis showed that both groups were predominantly classified as Enterotype 1 (Figure 1F). Furthermore, the relative abundance of the gut bacteria was compared at the phylum and genus levels between the ASD and TD groups (Figures 1G, H). Additionally, we explored the overall abundance differences across multiple microbial kingdoms; statistical analysis confirmed no significant differences in the relative abundances of bacteria, archaea, eukaryotes, and viruses between the two groups (nominal *p* > 0.05, Table S1). Collectively, these exploratory analyses suggest some differences in microbial community structure between ASD and TD children in this cohort, while α-diversity and overall microbial kingdom abundances were similar between groups
2. **Identification of gut bacterial species with differential abundance between ASD and TD groups**
  We performed LEfSe analysis to explore differences in the relative abundance of gut microbiota between Chinese children with ASD and TD. The analysis identified that *Lachnospira eligens* was higher in the ASD group (nominal *p* < 0.05; *LDA* > 3.5), while Bacteroidaceae, Ruminococcus, and *Phocaeicola plebeius* were higher in the TD group (nominal *p* < 0.05, *LDA* > 3.5; Figure 2A and Table S2). We also observed greater inter-individual heterogeneity in gut microbiota composition within the ASD group compared with the TD group (Bray–Curtis dissimilarity, *t*-test, nominal *p* < 0.01, Figure 2B). Using random forest (RF) analysis, we identified 10 bacterial species that contributed to the compositional differences between the two groups (Figure 2C). The RF model yielded an apparent AUC of 100% on the test set (Figure 2D), given the limited sample size, this perfect separation is likely due to overfitting and should not be interpreted as evidence of robust predictive performance. Taken together, these exploratory results suggest potential differences in gut microbiota composition between TD and ASD, but the findings are preliminary.
3. **Exploratory comparison of the gut microbiota co-occurrence network between Chinese children with ASD and TD**
  We performed an exploratory co-occurrence network analysis based on Spearman correlations to describe potential associations among gut microbial taxa. In both groups, the networks were primarily composed of genera from eight phyla: Bacillota, Bacteroidetes, Actinomycetota, Thermodesulfobacteriota, Synergistota, Lentisphaerota, Uroviricota, and Pseudomonadota (Figure 3). Positive correlations dominated in both networks, suggesting that cooperative rather than competitive interactions may prevail in this dataset. The network topology appeared more complex in the ASD group, with eight major interconnected modules, whereas the TD group displayed a simpler structure dominated by a single module. Quantitative network metrics showed an average degree of edges/nodes of 23.20 in ASD vs. 9.97 in TD. However, these descriptive differences should be interpreted with caution, as network parameters can be sensitive to sample size, sequencing depth, and correlation thresholds. In summary, co-occurrence network analysis identified observable structural and topological differences in microbial correlation patterns between children with ASD and TD in this cohort.
4. **Exploratory comparison of gut microbiota functions between Chinese children with ASD and TD**
  We performed an exploratory analysis of gut microbiome functions. A total of 2,779,403 and 2,842,378 genes were identified in the ASD and TD groups, respectively, with 2,461,832 shared genes (Figure 4A). Functional annotation based on the eggNOG database displayed distinct enrichment patterns of orthologous genes across groups. Genes associated with RNA processing and modification were relatively enriched in the ASD group. By contrast, functional categories including post-translational modification, chromatin structure and dynamics, cell wall/membrane/envelope biogenesis, cytoskeleton, carbohydrate transport and metabolism, and defense mechanisms were overrepresented within the TD group (Figure 4B). Analysis of carbohydrate-active enzyme (CAZy) genes suggested that Glycosyl Transferases were relatively more abundant in ASD, while Glycoside Hydrolases and Carbohydrate-Binding Modules were relatively more abundant in TD (Figure 4C). Using the CARD database, a higher number of antibiotic resistance genes was detected in the ASD group. Specifically, genes associated with glycopeptide resistance (e.g., vanY, vanH variant in the vanO cluster, vanG, and vanW variant in the vanB cluster) appeared enriched in ASD, whereas the TD group showed enrichment of adeF (fluoroquinolone and tetracycline resistance; Figure 4D). KEGG pathway analysis indicated that the most abundant functional categories in both groups were related to metabolism and environmental information processing. Nominal differences were observed for several pathways: for example, glycan biosynthesis and metabolism, environmental adaptation, development and regeneration, and transport and catabolism appeared enriched in TD, while xenobiotic biodegradation and metabolism appeared enriched in ASD (nominal *q* < 0.05; Figures 4E, F). Taken together, these exploratory functional analyses suggest potential differences in gut microbial gene composition and metabolic potential between ASD and TD children.
5. **Exploratory age-stratified comparison of gut microbiota between Chinese children with ASD and TD**
  We performed an exploratory age-stratified analysis based on cross-sectional data (age range 3–12 years). Sample sizes per age subgroup were small, especially in the ASD group: 3–5 y *n* = 7, 5–6 y *n* = 7, 6–7 y *n* = 5, 7–8 y *n* = 6, 8–9 y *n* = 3, 9–10 y *n* = 3, 10–12 y *n* = 3; TD: 3–5 y *n* = 8, 5–6 y *n* = 5, 6–7 y *n* = 9, 7–8 y *n* = 8, 8–9 y *n* = 4, 9–10 y *n* = 5, 10–12 y *n* = 5). A PCoA plot based on genus-level relative abundances showed a certain degree of separation between ASD and TD samples along axis 1 (PC1) and axis 2 (PC2), explaining 68.78% and 12.11% of the variability, respectively (Figure 5A). ANOSIM analysis in the 8–9 years subgroup (ASD *n* = 3, TD *n* = 4) suggested that inter-group differences might be larger than intra-group differences at several taxonomic levels (phylum to genus; Figure 5B). The Shannon index showed a nominal difference between ASD and TD only in the 8–9 years subgroup (Figure 5C). No significant differences were observed in other age subgroups. LEfSe analysis identified 26 age-associated microbial markers (Figure 5D). A heatmap of the top 35 genera suggested that the relative abundance of some taxa (e.g., Ruminococcus, Bilophila, Bacteroides, Faecalibacterium, Akkermansia, Clostridium) appeared to change with age in TD children, while such patterns were less evident in ASD (Figure 5E). However, because the data are cross-sectional, we cannot conclude that the gut microbiota “develops” or “changes” with age within individuals. Any apparent age-related trends may reflect cohort effects rather than true developmental trajectories.

## Discussion

Herein, our results revealed considerable differences in the composition, function, metabolic pathways, and microbial development of the gut microbiome between Chinese children with ASD and TD based on metagenomic sequencing analysis. Machine learning models were explored to assess the diagnostic potential of the gut microbiome in the ASD cohort, but these findings are preliminary and require independent validation. Therefore, monitoring gut microbiome changes of children with ASD in the early stages may be of interest for future research, but no clinical recommendation can be made at this stage. Recent studies have also highlighted the growing importance of identifying reliable biomarkers for ASD, as the heterogeneity of this disorder complicates early diagnosis.

The present study and prior research indicates that there is no significant difference in the alpha diversity of gut microbiota between children with ASD and TD (Chen et al., 2024), which is inconsistent with our previous 16S study results (Chen et al., 2024). However, different changes were observed in each age group. Specifically, in the 7–9-year-old age group, the gut microbiota diversity in children with ASD fluctuated drastically, showing a considerable decrease followed by a notable increase. Contrastingly, gut microbiota diversity in the TD group remained relatively stable, increased gradually, and eventually stabilized within the corresponding age group. Given the small sample sizes in age subgroups, these observations are descriptive and should not be overinterpreted. Analysis of the matched microbiota and age revealed that the gut microbiota in children with ASD did not exhibit a correlation with age, which might suggest developmental abnormalities but cannot be confirmed due to the cross-sectional design, this finding is partially consistent with previous studies (Wan et al., 2022). This observation aligns with recent research indicating that gut microbiota development in ASD may follow an atypical trajectory, potentially linked to underlying neurodevelopmental processes (Chen et al., 2024a). Through LEfse analysis, we identified a higher abundance of *L. eligens* and *Escherichia coli* in the gut of children with ASD, whereas *Bacteroidaceae, Prevotella, Ruminococcus*, and *Faecalibacterium prausnitzii* were under enriched. The nominal significance of these differences (*p* < 0.05, *LDA* > 3.5) was not adjusted for multiple comparisons; thus, these results are exploratory. Especially *Prevotella*, consistent with our previous findings (Li et al., 2023). It is possible that these different gut microbial species could potentially serve as candidate biomarkers for early ASD screening, but further validation is needed. For instance, *Ruminococcus, Lachnospiraceae*, and *Faecalibacterium* play a dominant role in pathways such as aromatic amino acid, L-serine, and glycine biosynthesis (Wan et al., 2022), which are important contributors to neurotransmitter synthesis (Pittard and Yang, 2008). The depletion of these related amino acid metabolic pathways might have detrimental effects on the host's mental health (Moore et al., 2000). Compared with TD children, those with ASD showed abnormal microbial functions, mainly concentrated in the amino acid and carbohydrate metabolism pathways. *Prevotella* produces succinate via polysaccharide utilization (Kovatcheva-Datchary et al., 2015). Succinate can bind to the succinate receptor GPR91 on the dendritic cell surface, enhancing the immune response of antigen-specific T cells and protecting the host's health (Ahrens et al., 2024; Rubic et al., 2008). However, children with ASD exhibit a certain degree of immune dysfunction (Ashwood et al., 2011). Thus, one might speculate that a decrease in metabolic pathways and substances related to neurotransmission may not suffice to sustain normal physiological and psychological development, but this hypothesis requires direct testing. The reduction in *Prevotella* in the gut of children with ASD could further exacerbate host immune dysfunction, but causal evidence is lacking.

Furthermore, the gut microbiota of children with ASD had a tendency toward lower expression of carbohydrate-active enzyme-related functional genes than that of TD children. This finding, if confirmed, might suggest certain problems in carbohydrate metabolism in children with ASD. Moreover, a higher proportion of children with ASD have been shown to have gastrointestinal disorders, such as diarrhea and constipation (Holingue et al., 2018). Additionally, a specific carbohydrate diet that requires strict avoidance and elimination of all grains, lactose, and sucrose-derived carbohydrates in the human diet (Haas and Haas, 1955) has good tolerance and meets the nutritional needs of children with ASD. After strict adherence to this diet for 16 weeks, the gastrointestinal health and behavior of patients with ASD improved (Barnhill et al., 2020). This result has been interpreted as indirect evidence that children with ASD may lack certain carbohydrate-metabolizing microbiota, which is consistent with our exploratory observation. However, this specific diet may further disrupt the gut microbiota of children with ASD because several microorganisms in the human gut rely on these carbohydrates for nutrition, thus worsening gastrointestinal issues in children with ASD.

Notably, analysis of drug-resistant genes revealed more resistance genes, especially for glycopeptide antibiotics, in the gut microbiome of children with ASD. While this finding may be associated with differential antibiotic exposure or other confounding factors, it does not and cannot indicate antibiotic overuse in this population. Further studies integrating clinical antibiotic prescription records are needed to clarify the underlying causes. Moreover, the use of antibiotics during infancy may be related to autism onset (Arrieta et al., 2014; Su et al., 2024). For instance, the period from birth to approximately the age of three is critical for human microbiota development. During this period, the use of antibiotics can disrupt the balance of the gut microbiota, affecting immune-mediated, metabolic, and neurological development (Arrieta et al., 2014). Early abuse of antibiotics can lead to the loss of typical microbial diversity and pathogen colonization in the human gut (Chen et al., 2017) and may result in long-term health problems such as inflammation, immune dysregulation, allergies, infections, and gastrointestinal diseases (Chen et al., 2017). Our results reveal an association between the abundance of antibiotic resistance genes (especially for glycopeptide antibiotics) and alterations in the gut microbiota composition in children with ASD. However, whether this reflects differential antibiotic exposure in early life or other underlying factors requires further investigation. Notably, a recent meta-analysis revealed significantly increased leptin levels and decreased progranulin concentrations in children with ASD, suggesting that peripheral metabolic factors, including adipokines, may also contribute to ASD pathophysiology (Chen et al., 2024b). These findings highlight the potential interplay between gut microbiota alterations, systemic metabolic dysregulation, and immune dysfunction in ASD, but remain speculative without direct mechanistic evidence.

The gut microbiota network has been previously shown to be crucial for host health because stable beneficial symbiotic bacteria and their related functions do not easily change over time (Lozupone et al., 2012). Our study found that the gut microbiota network in children with ASD appeared more complex than that in TD children, with more interactions between bacterial species (Li et al., 2023). However, network parameters are sensitive to sample size, correlation thresholds, and sequencing depth; therefore, this descriptive observation should be interpreted cautiously. Complex microbial community interactions are considered less stable (Coyte and Rakoff-Nahoum, 2019); gradually increasing the proportion of community member interactions almost always decreases the overall returns and stability (Coyte et al., 2015). Based on these assumptions, our findings could be interpreted as suggesting that the gut microbiota in patients with ASD may be characterized by an unstable and unfavorable ecosystem, but this remains a hypothesis. Recent evidence has demonstrated that peripheral systems, including the gut, can influence brain function through exosome-mediated intercellular communication (Chen et al., 2023). This finding provides a broader mechanistic framework for understanding how gut microbiota dysbiosis might contribute to ASD pathogenesis through the gut-brain axis.

This study has some limitations. First, the relatively small sample size may compromise statistical power and increase the risk of false-positive or false-negative results. Second, the sample size for age-subgroup analyses was extremely small (e.g., ASD 8–9 years *n* = 3), rendering any age-related findings purely descriptive and non-definitive. Third, although a multi-cohort meta-analysis of gut microbiota in children with autism spectrum disorder (ASD) demonstrated consistent and significant microbial differences between ASD and neurotypical control groups across studies (West et al., 2022), the geographical bias between the TD group and the ASD group may still restrict the generalizability of our findings to a broader population. Fourth, owing to data availability, only the metagenomic data of the gut microbiota was obtained and analyzed, lacking other data such as lifestyle and diet, medication, clinical indicators, physiological and biochemical markers, and metabolomics; Fifth, our KEGG pathway analysis was based only on a subset of significantly changed genes and did not include a formal contribution analysis linking the identified microbial biomarkers to specific pathways. Therefore, the functional interpretations should be considered exploratory, and future studies should employ methods such as stratified analysis or contribution analysis to validate these findings. Sixth, because the trajectory analysis was based on cross-sectional data rather than longitudinal follow-up, and because of potential age-related bias, it does not allow definitive conclusions regarding how the gut microbiota changes with age. Consequently, these limitations severely limit the depth of the present study, and the above results should be interpreted as preliminary and hypothesis-generating only. To address these limitations, future studies should include larger, longitudinal studies (necessary to properly investigate dynamic changes in the gut microbiota over time) and multi-center cohorts with diverse geographical representation to enhance generalizability, and should also employ formal contribution analysis to link microbial biomarkers to specific functional pathways.

In summary, our work provides exploratory evidence that children with ASD exhibit differences in gut microbiota composition, development, and gene function compared with TD children. However, due to the modest sample size, cross-sectional design, lack of external validation, and unmeasured confounders, the current data do not sufficiently support the conclusions that these differences can be used for non-invasive screening or that developmental abnormalities are definitively established. Our findings should be regarded as exploratory preliminary results, and independent validation in larger, well-controlled longitudinal cohorts is required before any translational application.

## Data Availability

The datasets presented in this study can be found in online repositories. The names of the repository/repositories and accession number(s) can be found in the article/Supplementary material.
