# Supplementary material for: Dysbiosis and unsustainable delayed gut microbiota development as non-invasive biomarkers for predicting autism spectrum disorder in Chinese children
Source: Front Microbiol. 2026 Jun 18;17:1753665. doi: 10.3389/fmicb.2026.1753665 (PMC13323027; doi:10.3389/fmicb.2026.1753665)
Supplement: Supplementary file 1 [file Table_1.docx]

**Table S1 Comparison of taxa between ASD and TD**

| **Taxa** | **ASD** | **TD** | **P value** |
| --- | --- | --- | --- |
| k__Archaea | 1.85E-05 ± 1.01E-05 | 8.38E-06 ± 1.31E-06 | 0.45 |
| k__Bacteria | 0.881 ± 0.0076 | 0.871 ± 0.005 | 0.29 |
| k__Eukaryota | 2.43E-05 ± 3.03E-06 | 5.13E-05 ± 2.80E-05 | 0.57 |
| k__Viruses | 0.022 ± 0.0053 | 0.023 ± 0.003 | 0.93 |
| Others | 0.097 ± 0.003 | 0.106 ± 0.002 | 0.02 |

ASD, autism spectrum disorders; TD, typically developing.
