# Supplementary material for: Dysbiosis and unsustainable delayed gut microbiota development as non-invasive biomarkers for predicting autism spectrum disorder in Chinese children
Source: Front Microbiol. 2026 Jun 18;17:1753665. doi: 10.3389/fmicb.2026.1753665 (PMC13323027; doi:10.3389/fmicb.2026.1753665)
Supplement: Supplementary file 2 [file Table_2.docx]

**Table S2 Comparison of the relative abundances of discriminant species (LDA score > 3.5) between the ASD and TD groups**

| **Taxa** | **ASD** | **TD** | **P value** |
| --- | --- | --- | --- |
| f__Bacteroidaceae | 0.1788 ± 0.0139 | 0.2489 ± 0.0157 | 0.0009 |
| g__Ruminococcus | 0.0051 ± 0.0012 | 0.0110 ± 0.0019 | 0.01 |
| s__Phocaeicola plebeius | 0.0014 ± 0.0004 | 0.0164 ± 0.0049 | 0.0009 |
| s__Lachnospira eligens | 0.0087 ± 0.0025 | 0.0027 ± 0.0005 | '0.0069 |

ASD, autism spectrum disorders; TD, typically developing.
